# High-fat diet-induced metabolic syndrome and oxidative stress in obese rats are ameliorated by yogurt supplementation

**DOI:** 10.1038/s41598-019-56538-0

**Published:** 2019-12-27

**Authors:** Shoumen Lasker, Md Mizanur Rahman, Faisal Parvez, Mushfera Zamila, Pintu Miah, Kamrun Nahar, Fariha Kabir, Surovi Binte Sharmin, Nusrat Subhan, Gias U. Ahsan, Md Ashraful Alam

**Affiliations:** 1 Department of Pharmaceutical Sciences, North South University, Dhaka, 1219 Bangladesh; 2 Department of Public Health, School of Health and Life Sciences, North South University, Dhaka, Bangladesh

**Keywords:** Animal disease models, Obesity

## Abstract

The main objective of this experiment was to determine the effects of yogurt supplementation on fat deposition, oxidative stress, inflammation and fibrosis in the liver of rats with high-fat (HF) diet-induced obesity. Male Wistar rats were used in this study and were separated into the following four different groups: the control, control + yogurt, high fat and high fat+ yogurt groups. The high fat groups received a HF diet for eight weeks. A 5% yogurt (w/w) supplement was also provided to rats fed the HF diet. Yogurt supplementation prevented glucose intolerance and normalized liver-specific enzyme activities in the HF diet-fed rats. Yogurt supplementation also significantly reduced the levels of oxidative stress markers in the plasma and liver of HF diet-fed rats. Moreover, inflammatory cell infiltration, collagen deposition and fibrosis in the liver of HF diet-fed rats were also prevented by yogurt supplementation. Furthermore, yogurt supplementation normalized the intestinal lining and brush border in HF diet-fed rats. This study suggests that yogurt supplementation potentially represents an alternative therapy for the prevention of metabolic syndrome in HF diet-fed rats.

## Introduction

Obesity is not only a disease but also a cause of many life-threatening diseases, including insulin resistance, oxidative stress and inflammation, hypertension and cardiovascular mortality^1,2^. The World Health Organization defines obesity as a “medical condition in which excess body fat is accumulated to the extent that it may have a negative effect on health”. This multifactorial disease develops because of a long-term imbalance between regular energy intake and energy expenditure^3^. According to a Chinese survey in the fields of nutrition and health, the incidence of overweight and obesity have significantly increased in adults in recent years^4,5^. According to another survey, the global prevalence of obesity has increased approximately two-fold in 2014 from the prevalence reported in 1980^6^. Mortality and morbidity have increased due to overweight rather than underweight^6^. In general, a high-fat (HF) diet induces the development of metabolic syndrome, which consists of oxidative stress, initiated atherogenic dyslipidemia, a pro-inflammatory and pro-thrombotic state, high blood pressure, central obesity and cardiovascular disease^1,7^.

Non-alcoholic fatty liver disease (NAFLD) is associated with a high calorie intake and many other diet-induced complications, such as metabolic syndrome and cardiovascular disease^8^. NAFLD is the most common chronic liver disease that develops in patients who do not abuse alcohol. A wide range of liver injuries is associated with NAFLD, ranging from simple steatosis to nonalcoholic steatohepatitis (NASH), advanced fibrosis and cirrhosis^9^. The signs and symptoms of metabolic syndrome may be induced in rats by feeding them diet rich in carbohydrates and fat^10^. The multifactorial disease NAFLD has many complex pathophysiologies, among which insulin resistance (IR), obesity and dyslipidemia are well known clinical markers^8,11^. Hepatic fat accumulation results from the synergistic effects of oxidative stress, hepatic lipid dysregulation and pro-inflammatory cytokines. A combination of various levels of dietary carbohydrates and fats from different sources, including nature, has been used in various NAFLD-related experimental studies^8,10^.

Yogurt is a milk product that results from the fermentation of lactic acid in milk by *Lactobacillus bulgaricus* and other lactic acid bacteria (LAB) species and is one of the well-known foods containing probiotics^12^. Probiotics may be defined as live microorganisms that exert some beneficial effects on the body of the host. Probiotics have many health-promoting effects, including the production of antimicrobial compounds, an improvement in immune functions and a decrease in the gut pH^13^. As shown in previous studies, a probiotic supplement may restore the microbiota in the gut and potentially represents an alternative therapy for irritable bowel syndrome^14,15^. Probiotics are also used to treat diabetes and hypercholesterolemia^16^. *Lactobacilli* and *Bifidobacteria* species may exert cholesterol-lowering effects on experimental models by degrading cholesterol and deconjugating bile salts^16,17^. A mixture of probiotics consisting of *Bifidobacterium* (*B. longum*, *B. lactis*, and *B. breve*) and *Lactobacillus* (*L. reuteri* and *L. plantarum*) decreased the lipid profiles in high-fat diet-fed rats^16^. A recent meta-analysis also showed beneficial effects of probiotics on subjects with NAFLD^18^. Other reports have also suggested beneficial effects of yogurt-containing probiotics on decreasing liver transferase activities, decreasing cholesterol levels and ameliorating NAFLD^19,20^.

However, the probiotic-mediated improvement in NAFLD and amelioration of oxidative stress in the liver were not explained properly. This study investigated the effects of a raw yogurt supplement on fat deposition, serum lipid levels, fatty liver, hepatic fibrosis and the intestinal environment in HF diet-fed rats to identify the beneficial effects of this functional food product.

## Results

### Effects of yogurt supplementation on body weight, food and water intake and calorie intake

Body weight gain is an important parameter for assessing the effect of a high-fat diet on the development of obesity and for monitoring its treatment. Initial and final body weights of the rats in different groups are presented in Table 1. High-fat diet-fed rats showed an increased body weight gain compared to the control rats. The final body weight of HF diet-fed rats was significantly increased (p < 0.05) compared to control rats. Yogurt supplementation in HF diet-fed rats significantly decreased the final body weight (p < 0.05) compared to the HF diet-fed rats. However, yogurt supplementation did not change the body weight of control rats.

**Table 1:** Effect of yogurt supplementation on body weight, food intake, water intake and total calorie intake in high fat diet fed in rats in comparison to control diet.

	Control	HF	Control + yogurt	HF + yogurt
Initial body weight (g)	193.23 ± 1.14_a_	185.52 ± 1.00_a_	189.30 ± 1.78_a_	184.38 ± 1.43_a_
Final body weight (g)	246.40 ± 2.96_a_	286.07 ± 3.78_b_	242.93 ± 2.92_a_	239.30 ± 4.11_a_
Food intake (g/day)	17.60 ± 1.06	16.61 ± 0.80	18.31 ± 1.08	16.10 ± 0.91
Water intake	23.96 ± 2.26	20.19 ± 1.84	20.35 ± 2.00	18.65 ± 1.48
Calorie intake (Kj/day)	322.2 ± 3.4_a_	462.2 ± 6.4_b_	330.1 ± 3.9_a_	447.1 ± 6.0_b_

Control rats were provided with control diet and HF rats were provided with HF diet. Yogurt was also supplied to control + yogurt and HF + yogurt groups. Data are presented as mean ± SEM. N = 6. Data analysis was conducted by one way ANOVA with Newman-Keuls post hoc test using Prism, version 7 software, for statistical analysis. Statistical significance was considered as p < 0.05 in all cases, here a vs b is significantly different at p < 0.05 level. All other non-marked groups are not significantly different.

Body weight gain mainly depends on the food and calorie intake in every animal species and must be recorded to obtain insights into the development of obesity. Thus, we measured the daily food and water intake of each rat. The average food and water intake of each rat are presented in Table 1. The average food and water intake among the groups did not change. However, HF diet-fed rats consumed significantly more calories (p < 0.05) than the control rats. Yogurt supplementation decreased the calorie intake of HF diet-fed rats; however, this decreased calorie intake was not significantly different from the HF diet-fed rats.

### Effect of yogurt on the wet weight of the liver and fat pad deposition

**Figure 1:**
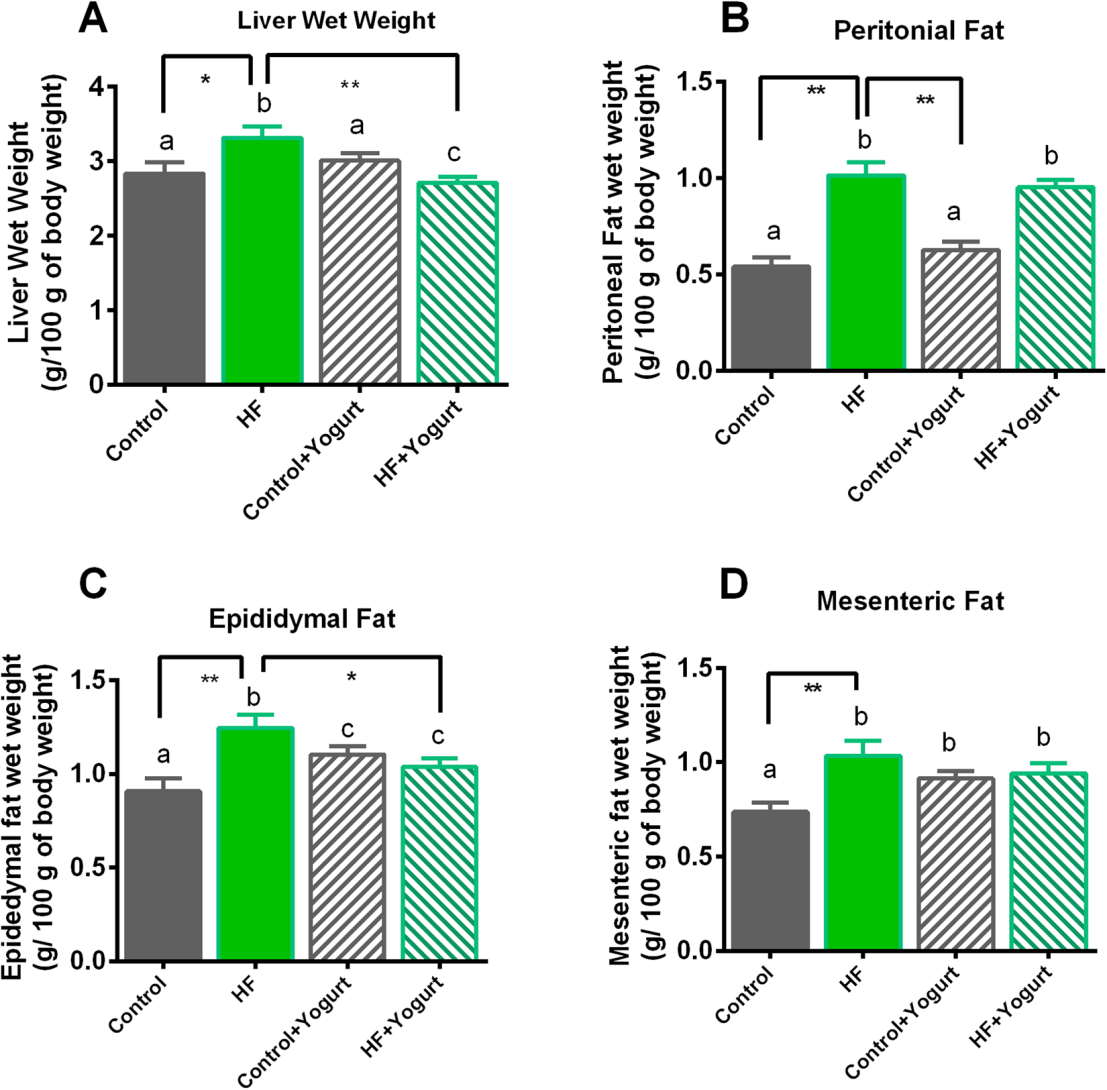
Effect of yogurt supplementation on liver wet weight (**A**), peritoneal (**B**), epididymal (**C**) and mesenteric (**D**) fat wet weight of high fat (HF) diet fed rats. Control rats were provided with control diet and HF rats were provided with HF diet. Yogurt was also supplied to control + yogurt and HF + yogurt groups. Data are presented as mean ± SEM, N = 6. Statistical analysis was performed by One Way ANOVA with Newman-Keuls post hoc test. Statistical significance is considered as p < 0.05. Asterisk (*) marked data are significantly different at p < 0.05 and (**) denotes p > 0.01.

Organ weights, especially the wet weights of the liver and fat, are important parameters for obtaining an understanding of diet-induced obesity. Wet weights of the liver from rats in each group are presented in Fig. 1. Wet weights of the liver were significantly increased in the HF group compared to the control rats (p < 0.05). Yogurt supplementation (5% w/w of the diet) decreased the wet weights of the liver in HF diet-fed rats (p < 0.05). Fat deposition in HF diet-fed rats is also presented in Fig. 1. The wet weight of peritoneal and mesenteric fat deposits was significantly increased in the HF group compared to the control group (p < 0.05). However, yogurt supplementation did not prevent peritoneal and mesenteric fat deposition compared to the HF diet-fed rats. Epididymal fat deposition was also significantly increased in HF diet-fed rats (p < 0.05) compared to control rats. The deposition of epididymal fat was significantly decreased by yogurt supplementation in HF diet-fed rats (p < 0.05).

### Effect of yogurt supplementation on the results of the oral glucose tolerance test (OGTT) in high-fat diet-fed rats

**Figure 2:**
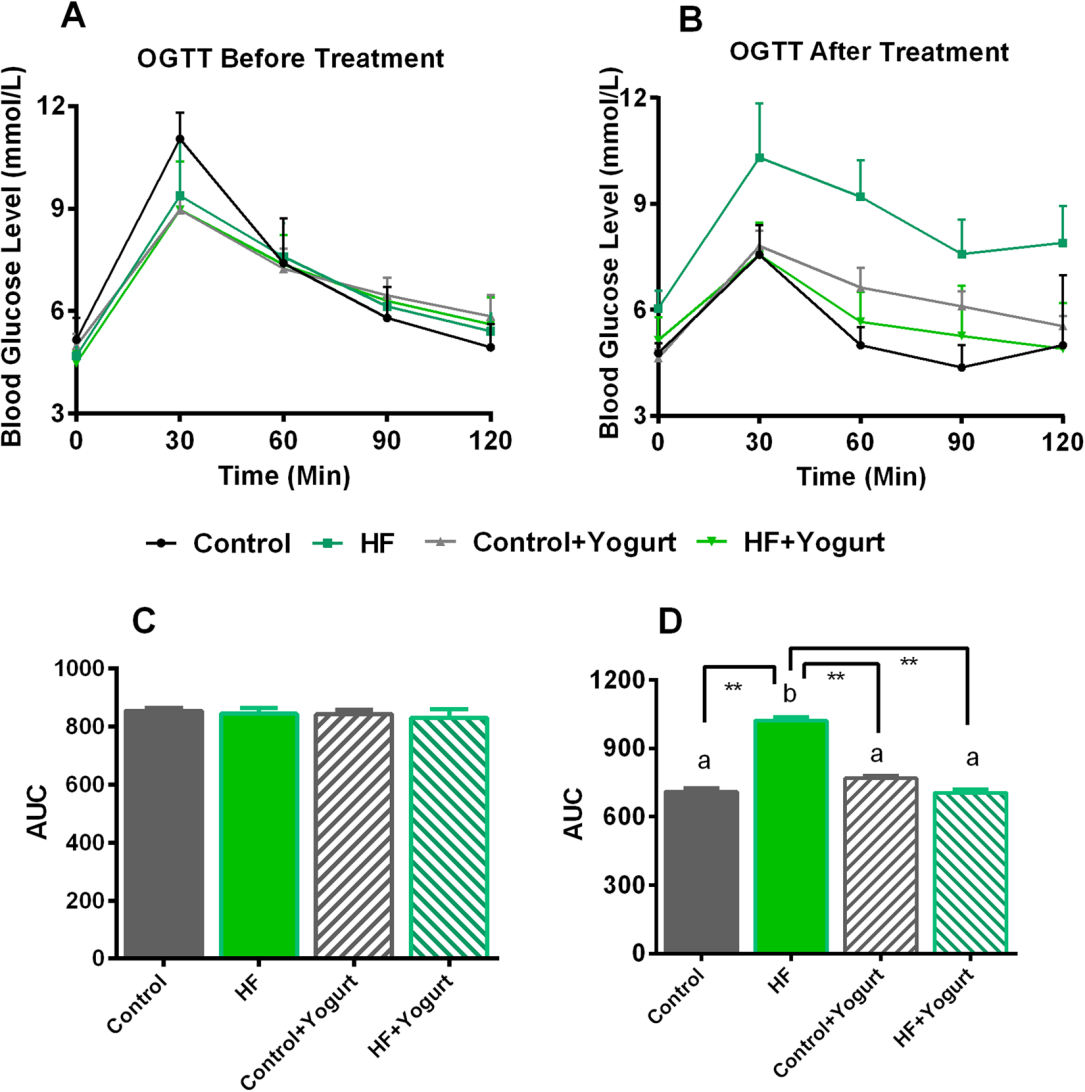
Effect of yogurt supplementation on oral glucose tolerance test (OGTT) before and after HF diet fed rats. Control rats were provided with control diet and HF rats were provided with HF diet. Yogurt was also supplied to control + yogurt and HF + yogurt groups. (**A**) OGTT has done at the beginning of the study and (**B**) OGTT done at the end of the study. The area under the curve (AUC) was determined, (**C**) OGTT done at the beginning (**D**) OGTT done at the end of the study. Data are presented as mean ± SEM. One way ANOVA followed by Newman-Keuls post hoc test was also conducted on AUC data and significance was considered as p < 0.05. Asterisk (*) marked data are significantly different at p < 0.05 and (**) denotes p > 0.01. N = 6. Also, a vs b is significantly different.

In obese subjects, a higher circulatory fasting glucose level in blood has been found. Therefore, the oral glucose tolerance test (OGTT) was performed to assess the ability of these rats to metabolize glucose after the consumption of a high-fat diet for 8 weeks. The results of the OGTT are presented in Fig. 2. At the beginning of the experiment, the basal glucose concentration in serum samples from the four groups was approximately 5 mmol/L (Fig. 2A). The glucose concentration in most of the groups increased at 30 min and decreased slowly to approximately the basal level up to 120 min after glucose administration. An analysis of the area under the curve (AUC) showed no significant differences in OGTT results among the groups tested (Fig. 2C).

**Figure 3:**
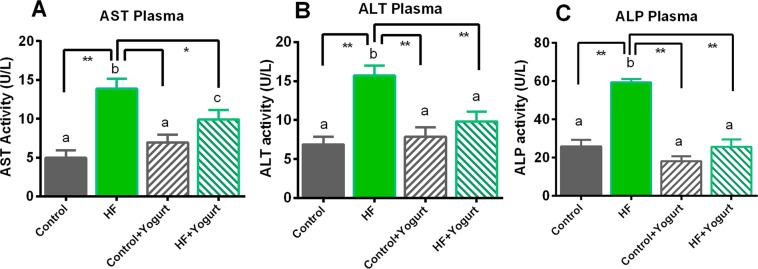
Effect of yogurt supplementation on liver marker (**A**) AST, (**B**) ALT and (**C**) ALP of HF diet rats. Control rats were provided with control diet and HF rats were provided with HF diet. Yogurt was also supplied to control + yogurt and HF + yogurt groups. Data are presented as mean ± SEM, N = 6. Statistical analysis was performed by One Way ANOVA with Newman-Keuls post hoc test. Statistical significance is considered as p < 0.05. Asterisk (*) marked data are significantly different at p < 0.05 and (**) denotes p > 0.01. Also, a vs b and c vs a or b are significantly different.

After rats had consumed the high-fat diet for 56 days, the basal glucose level was similar in the 4 groups and rose at 30 min after glucose administration. However, the serum glucose level was normalized within 60–120 min in most of the groups tested, except the high-fat diet-fed rats. After 120 min, HF diet-fed rats showed high blood glucose level, whereas, yogurt supplementation significantly reduced the blood glucose level in HF + yogurt group (Fig. 3B). The AUC analysis revealed significant differences between control and HF diet-fed rats (p < 0.05) (Fig. 2D). Yogurt supplementation significantly normalized the AUC value of HF diet-fed rats (p < 0.05) (Fig. 2D). Yogurt supplementation did not change the AUC of control rats.

### Effects of yogurt supplementation on serum alanine aminotransferase (ALT), aspartate aminotransferase (AST) and alkaline phosphatase (ALP) activities in HF diet-fed rats

High-fat diet-fed animals showed fat deposition in the liver and eventually developed steatosis and hepatic damage. Hepatic damage was assessed by measuring the activities of enzyme markers of liver function. Serum ALT, AST and ALP activities are increased when hepatic damage occurs, and we measured the activities of these enzymes in control and high-fat diet-fed animals. Changes in liver enzyme functions are shown in Fig. 3. Plasma AST activity was significantly increased (p < 0.05) in HF diet-fed rats compared to the control rats. Oral supplementation with yogurt for eight weeks normalized the AST activities in the HF + yogurt group (p < 0.05) compared to HF diet-fed rats. Plasma ALT and ALP activities were also increased 2–3-fold in HF diet-fed rats compared to the control group (p < 0.05). Oral supplementation with yogurt also normalized the activities of these liver enzymes by decreasing the ALT and ALP activities in the rats in the HF + yogurt group (p < 0.05) compared to HF diet-fed rats. However, yogurt supplementation did not alter the activities of these enzymes in control rats.

### Effects of yogurt supplementation on plasma and liver levels of oxidative stress markers in HF diet-fed rats

**Figure 4:**
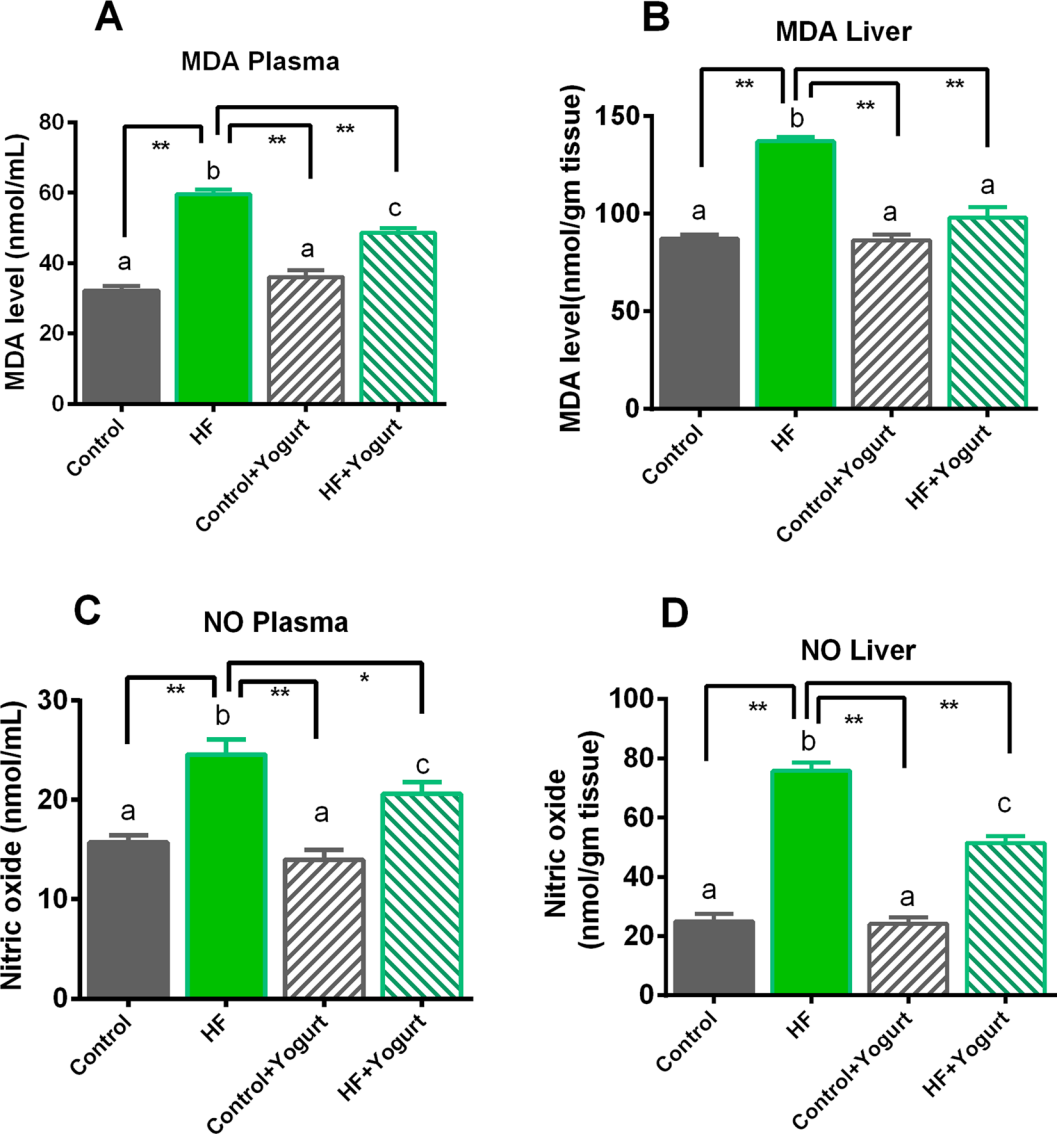
Effect of yogurt supplementation on oxidative stress marker (**A**) MDA plasma, (**B**) MDA liver (**C**) NO plasma and (D) NO liver of HF diet rats. Control rats were provided with control diet and HF rats were provided with HF diet. Yogurt was also supplied to control + yogurt and HF + yogurt groups. Control rats were provided with control diet and HF rats were provided with HF diet. Yogurt was also supplied to control + yogurt and HF + yogurt groups of rats. Data are presented as mean ± SEM, N = 6. Statistical analysis was performed by One Way ANOVA with Newman-Keuls post hoc test. Statistical significance is considered as p < 0.05. Asterisk (*) marked data are significantly different at p < 0.05 and (**) denotes p > 0.01. Also, a vs b and c vs a or b are significantly different.

Oxidative stress is assessed by measuring malondialdehyde (MDA), nitric oxide (NO) and advanced protein oxidation product (APOP) concentrations and myeloperoxidase (MPO) activities in the plasma and liver. Lipid peroxidation and MDA formation in different groups are shown in Fig. 4. Rats that consumed the HF diet exhibited significantly increased lipid peroxidation (p > 0.05) compared to the control rats. Yogurt supplementation prevented the lipid peroxidation in HF diet-fed rats (Fig. 4). Moreover, yogurt supplementation did not affect the MDA levels in control rats (Fig. 4).

Nitric oxide is another crucial component contributing to the development of nitrosative stress in high-fat diet-fed animals. The NO data are presented in Fig. 4. In the present study, the NO concentrations in plasma and liver were also significantly increased in HF diet-fed rats compared to control rats (p > 0.05). Yogurt supplementation (5% w/w of the diet) significantly (p<0.05) decreased the NO levels in the plasma and liver in HF diet-fed rats (Fig. 4). However, the NO levels in the plasma and liver were not affected in control rats received the yogurt supplementation (Fig. 4).

**Figure 5:**
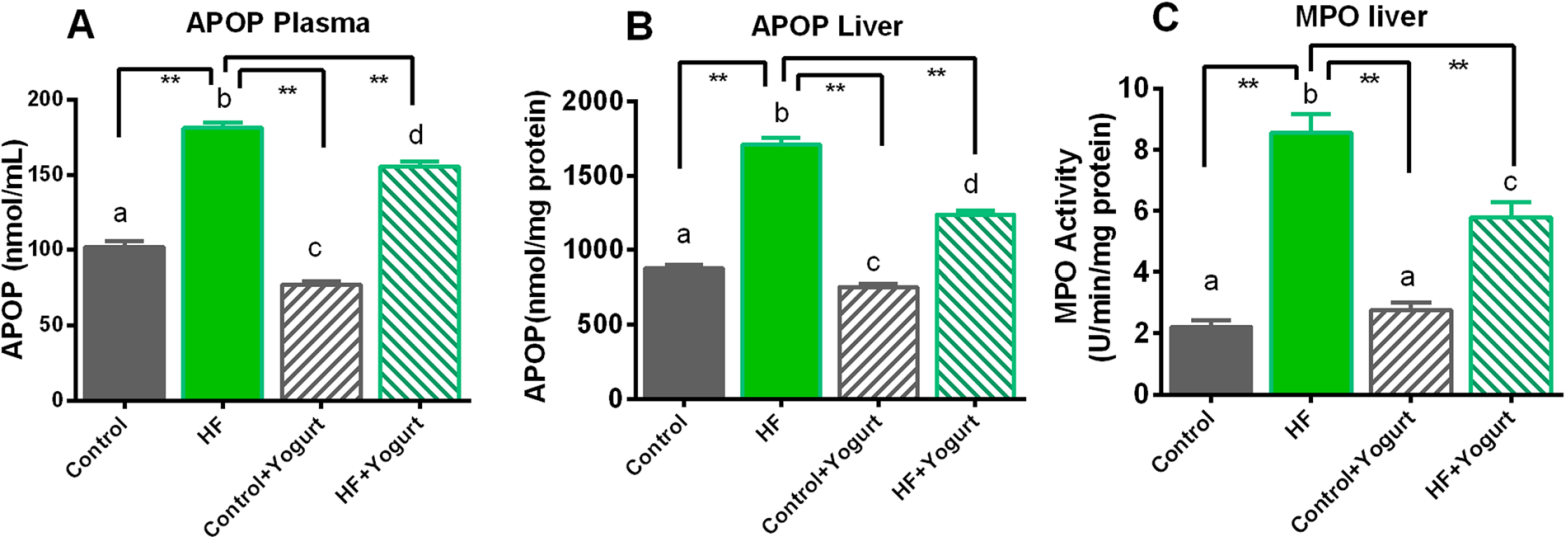
Effect of yogurt supplementation on oxidative stress marker (**A**) APOP plasma, (**B**) APOP liver and (**C**) MPO liver of HF diet rats. Control rats were provided with control diet and HF rats were provided with HF diet. Yogurt was also supplied to control + yogurt and HF + yogurt groups. Data are presented as mean ± SEM, N = 6. Statistical analysis was performed by One Way ANOVA with Newman-Keuls post hoc test. Statistical significance is considered as p < 0.05. Asterisk (*) marked data are significantly different at p < 0.05 and (**) denotes p > 0.01. Also, a vs b and c vs a or b; and d vs c, b or a are significantly different.

APOP concentrations were also significantly increased in HF diet-fed rats compared to control rats (P > 0.05) (Fig. 5). Yogurt supplementation significantly decreased APOP levels (P > 0.05) in high-fat diet-fed rats (Fig. 5). Yogurt supplementation also lowered APOP levels both in plasma and liver of control rats significantly (P < 0.05) (Fig. 5).

Myeloperoxidase (MPO) is a component of mononuclear cells that participates in oxidative stress when these cells infiltrate tissues such as the liver. Thus, MPO is considered another marker of oxidative stress and inflammation. In this study, MPO activity was significantly increased in the liver of HF diet-fed rats (p > 0.05) compared to the control rats. Yogurt supplementation decreased MPO activities in HF diet-fed rats to near normal levels compared to the HF diet-fed rats (Fig. 5).

### Effects of yogurt supplementation on antioxidant enzyme and glutathione levels in HF diet-fed rats

**Figure 6:**
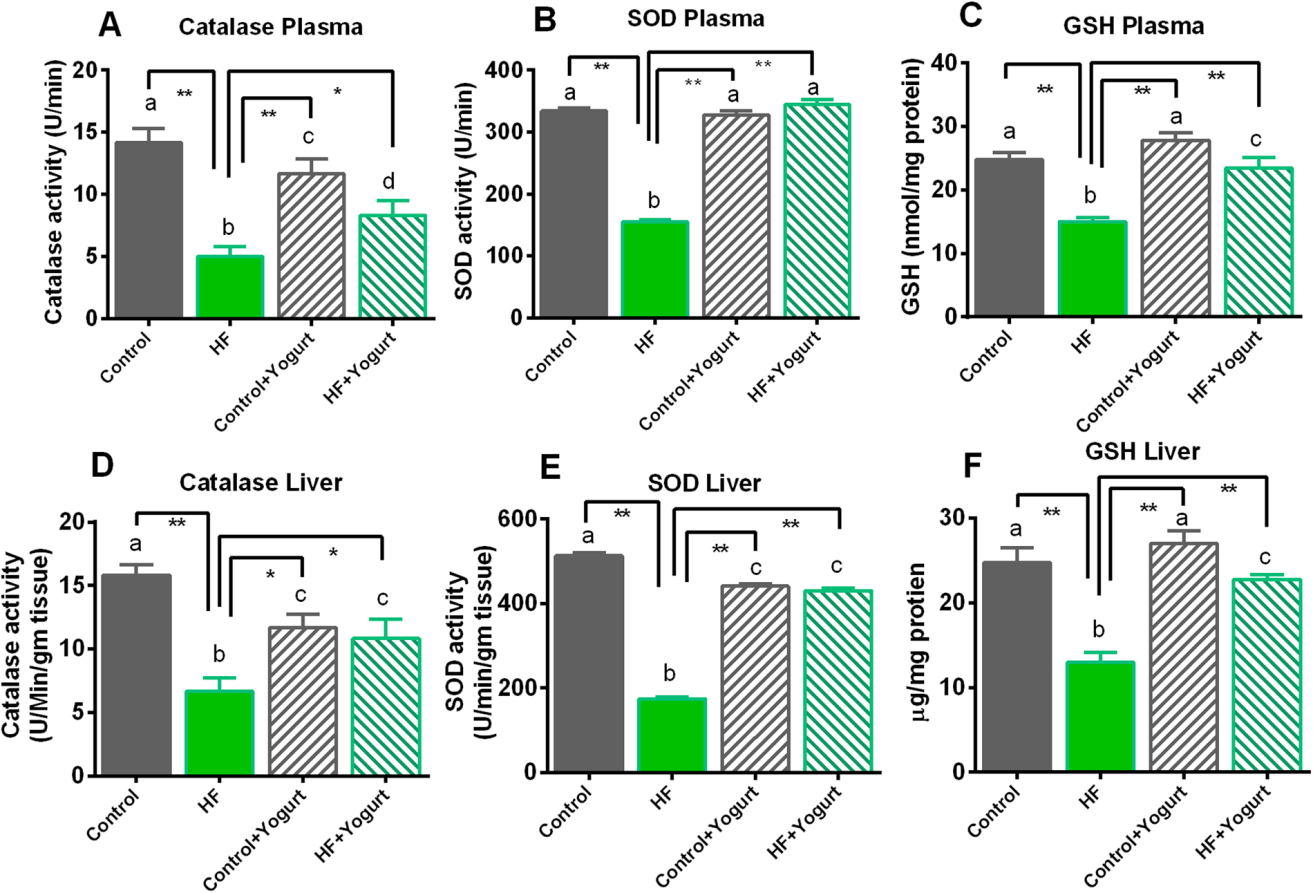
Effect of yogurt supplementation on cellular antioxidants (**A**) catalase plasma, (**B**) SOD plasma, (**C**) GSH plasma, (**D**) catalase liver, (**E**) SOD liver and (**F**) GSH liver of HF diet rats. Control rats were provided with control diet and HF rats were provided with HF diet. Yogurt was also supplied to control + yogurt and HF + yogurt groups. Data are presented as mean ± SEM, N = 6. Statistical analysis was performed by One Way ANOVA with Newman-Keuls post hoc test. Statistical significance is considered as p < 0.05. Asterisk (*) marked data are significantly different at p < 0.05 and (**) denotes p > 0.01. Also, a vs b and c vs a or b; and d vs c, b or a are significantly different.

Superoxide dismutase (SOD), catalase and reduced glutathione (GSH) are naturally produced cellular antioxidants that are responsible reducing oxidative stress. These cellular antioxidant activities were compromised due to the increase in oxidative stress in HF diet-fed rats, and the data are presented in Fig. 6. In this study, SOD activity was significantly decreased (p < 0.05) in HF diet-fed rats compared to the control group. SOD activity in both the plasma and liver was significantly (p < 0.05) restored by yogurt supplementation in the HF + yogurt group (Fig. 6) compared to HF diet-fed rats.

Catalase is another tissue antioxidant enzyme that converts hydrogen peroxide to water. In the present study, catalase activity was significantly decreased in HF diet-fed rats (p < 0.05) compared to the control rats (Fig. 6). Yogurt supplementation significantly restored the catalase activity in HF diet-fed rats (p < 0.05) to approximately the levels of control rats (Fig. 6). Yogurt supplementation altered the catalase activity compared to control rats however, the catalase activity was higher than the HF diet fed rats (Fig. 6).

The concentrations of reduced glutathione (GSH) in the plasma and liver were also decreased in HF diet-fed rats compared to the control group (p < 0.05). Yogurt supplementation significantly increased the GSH concentrations in the plasma and liver of HF diet-fed rats (p < 0.05). Moreover, yogurt supplementation did not change the GSH concentration in control rats (Fig. 6).

### Effect of yogurt supplementation on the lipid profiles of HF diet-fed rats

**Figure 7:**
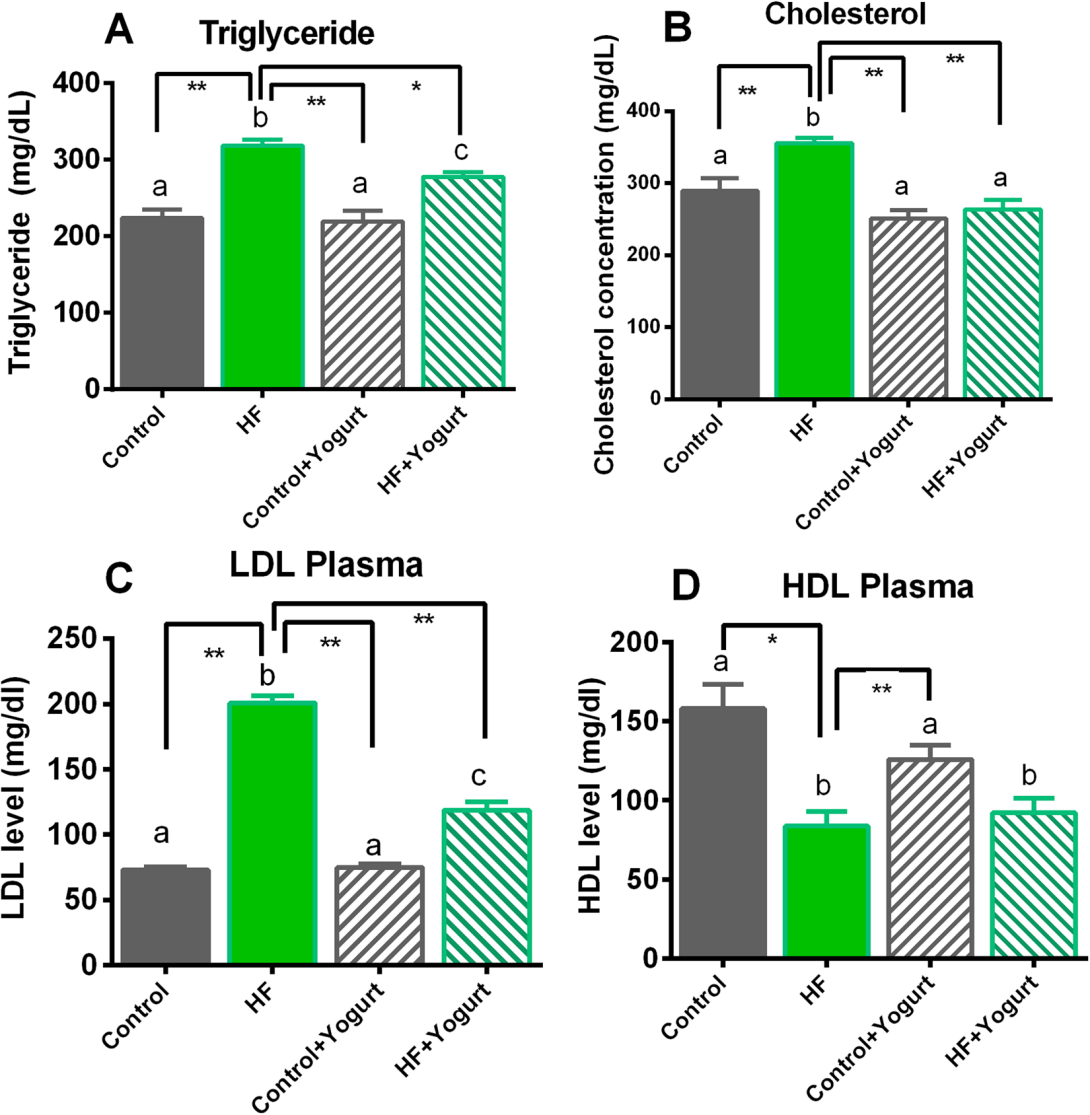
Effect of yogurt supplementation on lipid profiles of HF diet fed rats. Control rats were provided with control diet and HF rats were provided with HF diet. Yogurt was also supplied to control + yogurt and HF + yogurt groups. Data are presented as mean ± SEM, N = 6. Statistical analysis was performed by One Way ANOVA with Newman-Keuls post hoc test. Statistical significance is considered as p < 0.05. Asterisk (*) marked data are significantly different at p < 0.05 and (**) denotes p > 0.01. Also, a vs b and c vs a or b are significantly different.

We measured the level of total cholesterol and triglyceride levels in the plasma of HF diet-fed rats to evaluate the lipid-lowering effect of yogurt supplementation on high-fat diet-fed rats. The lipid profile of rats that received the yogurt supplement and HF diet is presented in Fig. 7. In the present study, plasma triglyceride and total cholesterol levels were significantly increased (p < 0.05) in rats that consumed the HF diet compared to the control rats. Yogurt supplementation significantly reduced the plasma triglyceride and total cholesterol levels in HF diet-fed rats (p < 0.05) (Fig. 7). Moreover, the low density lipoprotein (LDL) cholesterol level was also significantly increased (p < 0.05) in HF diet-fed rats compared to the control rats. Yogurt supplementation significantly decreased the plasma LDL cholesterol level in HF diet-fed rats (p < 0.05) (Fig. 7). Furthermore, the high density lipoprotein (HDL) cholesterol level was significantly decreased in high-fat diet-fed rats (p < 0.05) compared to control rats, and yogurt supplementation did not produce further improvements (Fig. 7). However, no changes were observed in the lipid profiles of control rats receiving the yogurt supplement (Fig. 7).

### Histological assessment of the liver and intestine

**Figure 8:**
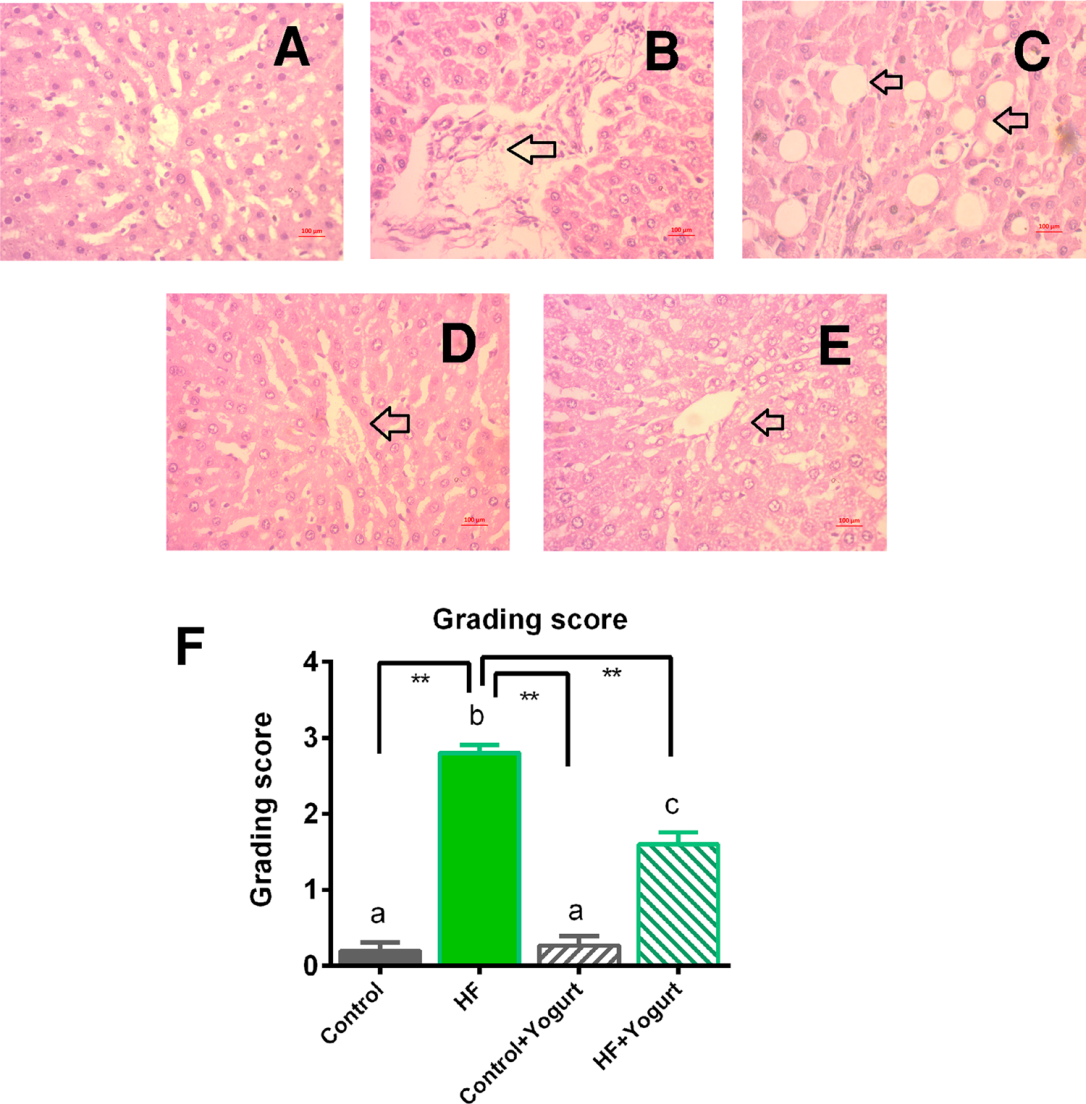
Effect of yogurt supplementation on liver inflammation in HF diet fed rats. Control rats were provided with control diet and HF rats were provided with HF diet. Yogurt was also supplied to control + yogurt and HF + yogurt groups. (**A**) Control, liver section showed normal histological structure without any lesion and necrosis. (**B**,**C**)- HF, showed fat droplet deposition and inflamed area in liver section. (**D**) Control + yogurt, showed similar histological structure as in control rats. (**E**) HF + yogurt, showed improvement in reducing inflammation and fat droplet deposition in liver section. F- hepatic lesion grading score in various groups. Magnification is X40. Data are presented as Mean ± SEM. Statistical analysis was done by One way ANOVA followed by Newman Keuls test using Prism Software. Statistical significance was considered at p < 0.05 in all cases. Asterisk (*) marked data are significantly different at p < 0.05 and (**) denotes p > 0.01. Also, a vs b and c vs a or b are significantly different.

**Figure 9:**
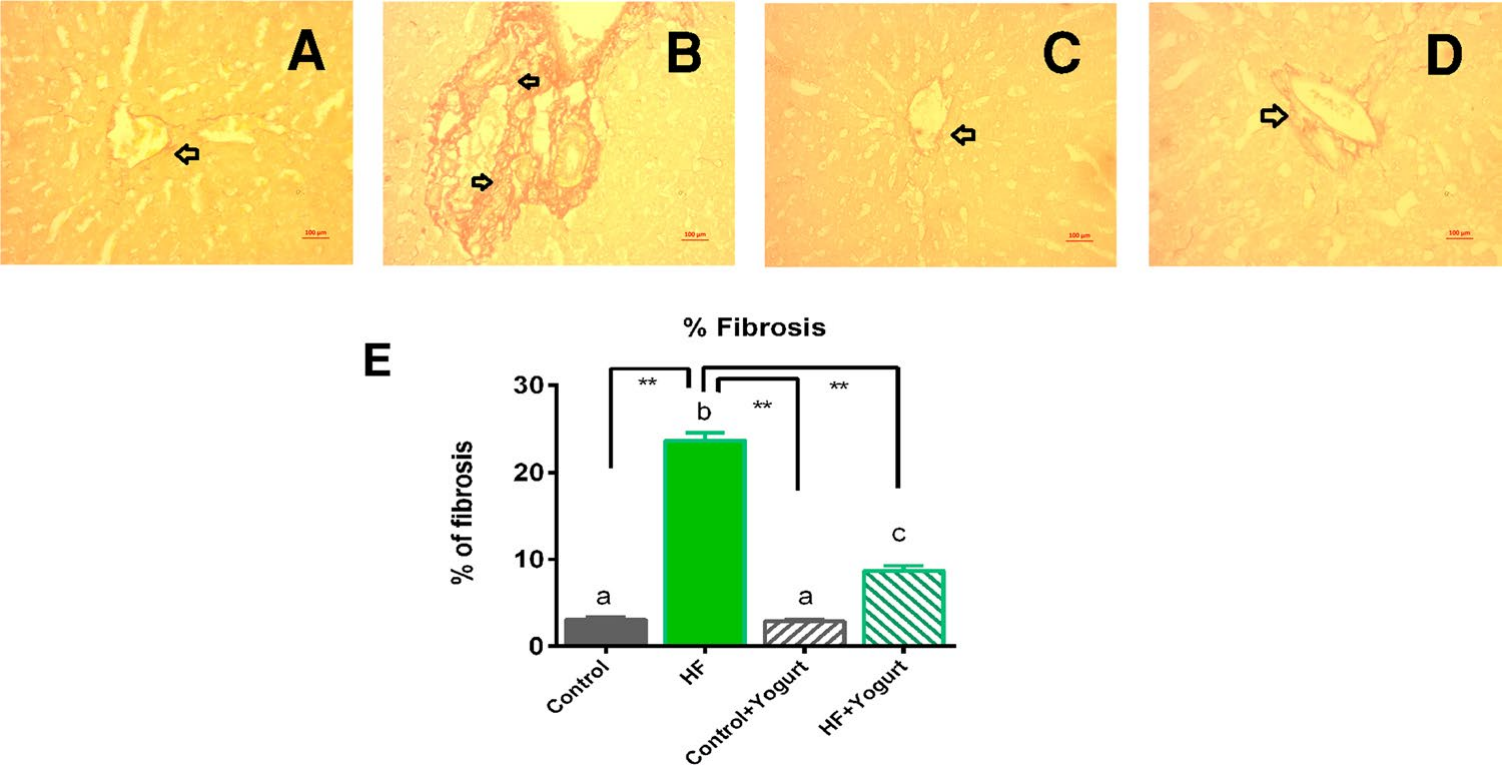
Effect of yogurt supplementation on liver fibrosis in HF diet fed rats. Control rats were provided with control diet and HF rats were provided with HF diet. Yogurt was also supplied to control + yogurt and HF + yogurt groups. (**A**) Control, showed normal baseline collagen present in liver section. (**B**) HF, showed collagen deposition and fibrosis as marked as red zone in the liver section. (**C**) Control + yogurt, showed similar collagen distribution as in control rats. (**D**) HF + yogurt, significant improvement has been seen in reducing the collagen deposition and fibrosis in liver section. (**E**) represents the percent of fibrosis in liver tissues in various group of rats. Magnification is X40. Data are presented as Mean ± SEM. Statistical analysis was done by One way ANOVA followed by Newman Keuls test using Prism Software. Statistical significance was considered at p < 0.05 in all cases. Asterisk (*) marked data are significantly different at p < 0.05 and (**) denotes p > 0.01. Also, a vs b and c vs a or b are significantly different.

**Figure 10:**
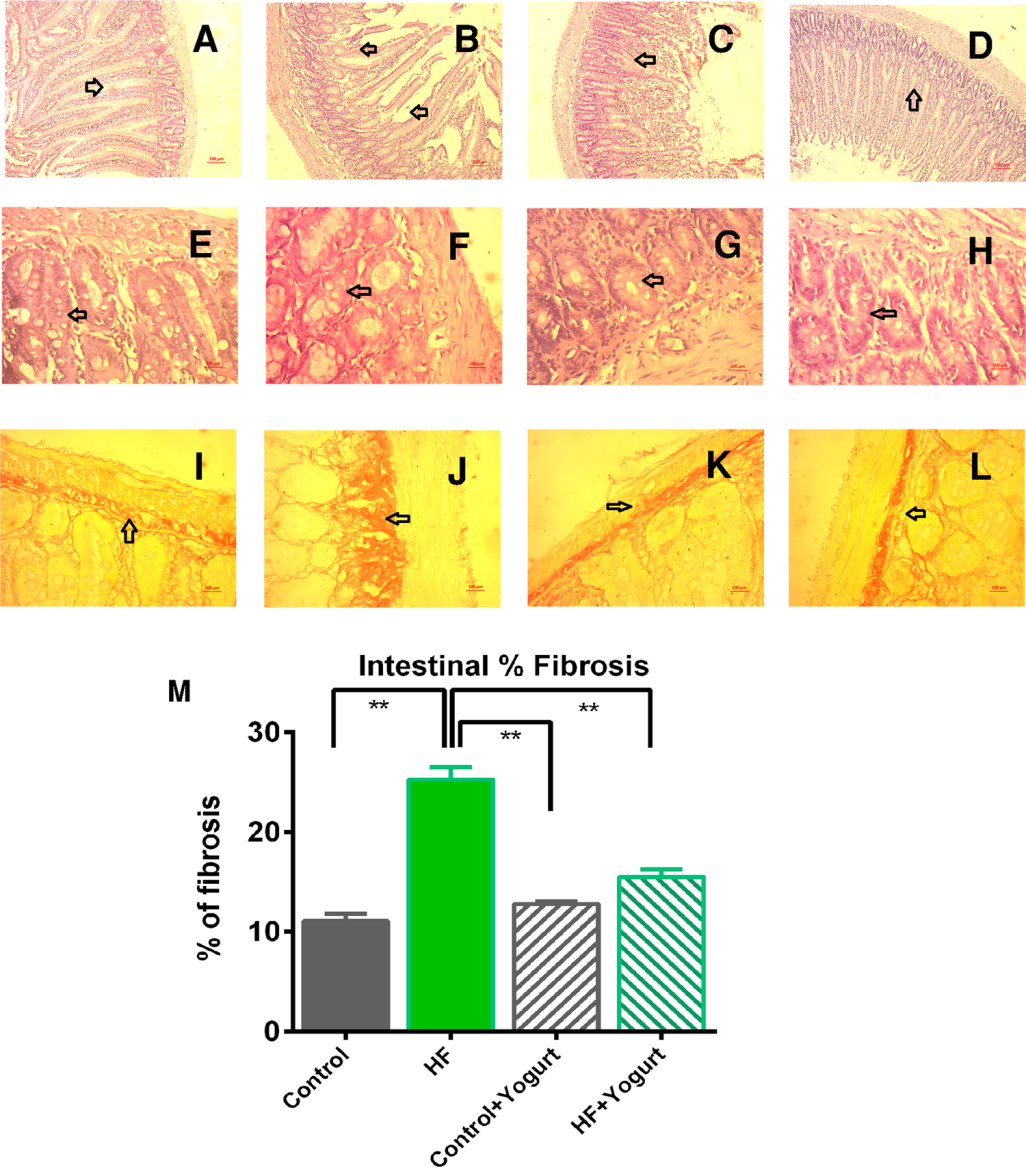
Effect of yogurt supplementation on intestinal cryptic and villi structure as well as fibrosis in intestinal basement layer in HF diet fed rats. Control rats were provided with control diet and HF rats were provided with HF diet. Yogurt was also supplied to control + yogurt and HF + yogurt groups. (**A**), E- Control, showed normal villi and crypt region in the intestine. (**B**), F- HF, showed distorted villi and crypt region in the intestine. (**C**), G- Control + yogurt, showed normal villi and crypt region in the intestine. (**D**), H- HF + yogurt, yogurt supplementation improved the villi and crypt region improvement in high fat diet fed rats. First upper panel magnification is X10 and other panels magnification are X40. I-control, showed normal distribution of collagen in the intestinal basement. J- HF, showed collagen deposition and fibrosis in the intestinal basement. K- Control + yogurt, showed normal distribution of collagen in the intestinal basement as in control rats. L- HF + yogurt, showed reduced collagen deposition and fibrosis in the intestinal basement. M, the % of fibrosis in the intestinal basement. Data are presented as Mean ± SEM. Statistical analysis was done by One way ANOVA followed by Newman Keuls test using Prism Software. Statistical significance was considered at p < 0.05 in all cases. Asterisk (*) marked data are significantly different at p < 0.05 and (**) denotes p > 0.01.

**Figure 11:**
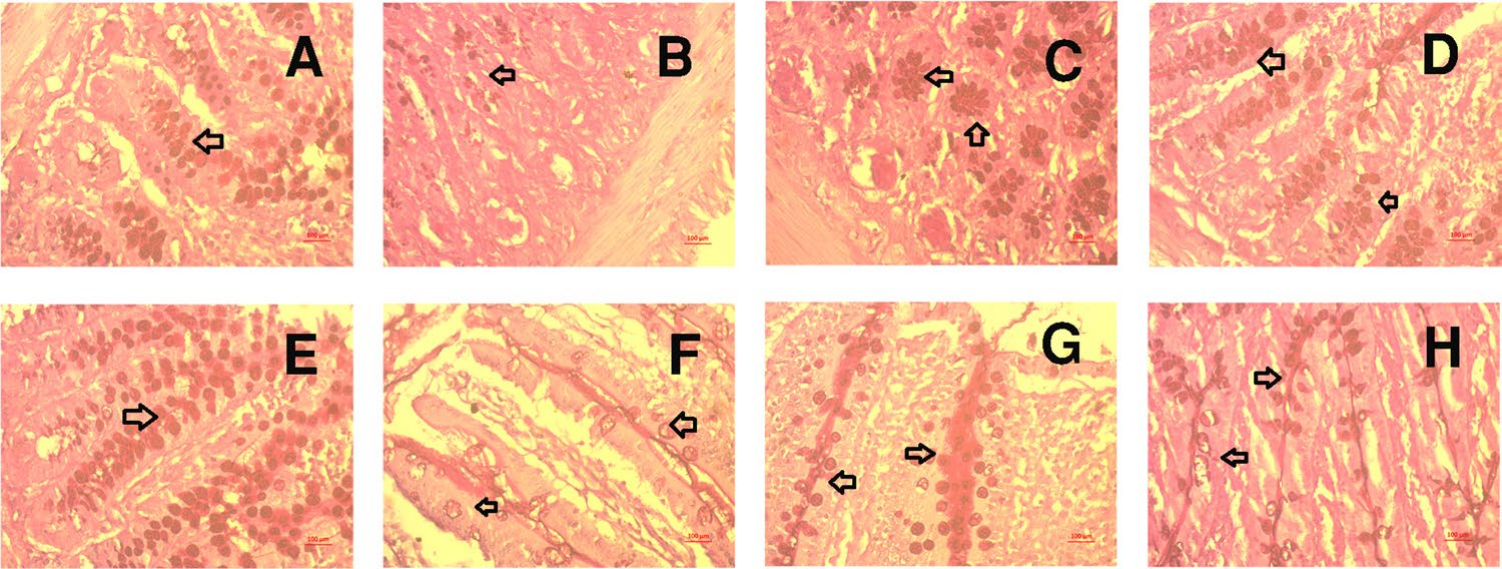
Effect of yogurt supplementation on goblet cell population in the crypt and villi region of intestine in HF diet fed rats (Periodic Acid Schiff Staining). Control rats were provided with control diet and HF rats were provided with HF diet. Yogurt was also supplied to control + yogurt and HF + yogurt groups. (**A**), E- Control, showed normal distribution of goblet cells population in the crypt and villi region of intestine. (**B**), F- HF, showed loss of goblet cells population in the crypt and villi region of intestine; (**C**), G- Control + yogurt, showed similar distribution of goblet cells population in the crypt and villi region of intestine as in control rats. (**D**), H- HF + yogurt, showed reappearance and increased number of goblet cells population in the crypt and villi region of intestine. Magnifications are X40.

Photomicrographs of liver tissues from all experimental groups are presented in Figs. 8–11. In this study, the hepatic tissue from the control group revealed a normal architecture of hepatocytes, with no appearance of lipid/fat deposition and inflammatory cell infiltration (Fig. 8), whereas HF diet-fed groups showed degenerative changes in hepatocyte along with lipid/fat droplet deposition and inflammatory cell infiltration (Fig. 8). HF diet-fed rats that received the yogurt supplement retained the normal architecture of hepatocytes, displayed less fat/lipid deposition and inflammatory cell infiltration, and showed a similar pattern to the control group, indicating the hepatoprotective effect of yogurt supplementation following HF diet feeding (Fig. 8). The steatosis and inflammation in the liver were assessed and graded with numbers. HF diet feeding in rats significantly increased the steatosis and inflammation grades (p < 0.05) compared to control rats. Yogurt supplementation significantly reduced the scores for steatosis and inflammation in HF diet-fed rats (P < 0.05) (Fig. 8F).

Sirius red staining was performed in this study to evaluate the effect of yogurt on HF diet-induced liver fibrosis. Liver fibrosis was more severe in the HF diet-fed group compared to control rats (Fig. 9). The control group and control + yogurt group showed much less fibrosis, whereas the co-administration of yogurt with the HF diet reduced the hepatic fibrosis in the HF diet-fed group, suggesting that yogurt supplementation significantly prevented HF diet-induced hepatic fibrosis (Fig. 9). The percentage of fibrosis was also significantly increased in HF diet-fed rats (p < 0.05) compared to the control rats (Fig. 9E). Yogurt supplementation significantly reduced the percentage of fibrosis in HF diet-fed rats (Fig. 9E).

Staining was performed to assess the intestinal histology, particularly the microvilli and crypts. High-fat diet feeding in rats was associated with a distorted microvilli structure and disoriented crypts compared to the control rats (Fig. 10). Yogurt supplementation restored the structures of the microvilli and crypts and increased the number of goblet cells per crypt in HF diet-fed rats (Fig. 10). Moreover, HF diet-fed rats showed increased collagen fiber deposition in the intestinal basement membrane compared to the control rats (Fig. 10). Yogurt supplementation normalized the collagen fiber deposition in high-fat diet-fed rats (Fig. 10).

Periodic acid Schiff (PAS) staining of the intestine revealed a substantial number of mucus-producing goblet cells both in crypts and villi of the control rats (Fig. 11). HF diet feeding in rats decreased the number of goblet cells, and this change was reversed by yogurt supplementation (Fig. 11).

Moreover, Prussian blue staining showed the accumulation of free iron droplets in HF diet-fed rats compared to the control rats (Supplemental Fig. 1). Yogurt supplementation prevented the iron deposition in the liver of high-fat diet-fed rats (Supplemental Fig. 1).

## Discussion

Obesity-related health complications are increasing in various societies worldwide. The consumption of a high-fat and high-energy diet is considered a major cause of the development of these complications. As shown in the present study, rats that consumed a high-fat diet developed glucose intolerance, dyslipidemia and oxidative stress in the liver. Moreover, yogurt supplementation prevented the glucose intolerance and decreased plasma lipid levels in high-fat diet-fed rats. Yogurt supplementation also prevented oxidative stress and decreased lipid peroxidation in the plasma and liver by restoring the cellular antioxidants.

Rats that consumed a high-fat diet exhibited increased plasma glucose levels and plasma lipid levels, including cholesterol and triglyceride levels. The saturated fats present in the high-fat diet are responsible for the increase in the glucose and lipid profiles^21^. According to previous studies, rat fed a high-fat diet display increased serum glucose concentrations^22^. Rats fed a high-fat diet also developed glucose intolerance and were unable to properly utilize glucose to establish homeostasis after a glucose challenge. In fact, the gram-negative bacterial cell wall contains lipopolysaccharide (LPS), which may penetrate the gut due to the leakiness of intestinal mucosa caused by a high fat content in the diet^23^. Researchers have postulated that bacterial LPS may cause endotoxemia and inflammation^24^. Intestinal permeability may also facilitate the entry of bacterial fragments into the body and subsequent interaction with the Toll-like receptor to activate innate and adaptive immunity and cause hyperglycemia and insulin resistance^25^. However, *Lactobacilli* species are capable of strengthening the epithelial barrier and may potentially prevent LPS-mediated inflammation and hyperglycemia^26^. Yogurt supplementation improved the glucose utilization in this experiment, as evidenced by the results from the OGTT, and these results are supported by a previous experiment^27^. Based on the current investigation, yogurt supplementation also prevented the deposition of epididymal adipose tissue in high-fat diet-fed rats. Yogurt also significantly decreased the risk of metabolic syndrome, including obesity, in previous studies^28,29^.

Oxidative stress caused by the consumption of a high-fat diet is evident in most experimental models and patients with clinical conditions^30,31^. As shown in the present study, the levels of the lipid peroxidation product malondialdehyde, nitric oxide and advanced protein oxidation products were increased in high-fat diet fed rats. The increase in oxidative stress parameters might be associated with the decreased SOD and catalase activities observed in the present study. These results are consistent with previous studies showing that tissue antioxidant defenses may be compromised in high-fat diet-fed animals^10,32^. Yogurt supplementation prevented the oxidative stress and restored the tissue antioxidants in high-fat diet-fed rats in our study. Probiotic supplementation may enhance the antioxidant defenses in tissues experiencing oxidative stress. Lactic acid bacteria-rich pickled Chinese cabbage supplemented with a HFD increases the SOD and GSH-Px activities in ICR mice^33^. Moreover, probiotics positively modulate free radical metabolism by increasing the activities of antioxidant enzymes and decreasing the concentrations of malondialdehyde (MDA) and nitric oxide^34,35^.

Oxidative damage in tissues also leads to hepatocyte damage in high-fat diet-fed rats, as evidenced by the increased plasma activities of the AST, ALT and ALP enzymes. These enzymes are considered markers of hepatic dysfunction. Generally, hepatocyte damage causes these enzymes to be transported to the plasma. High-fat diet-fed rats showed increased plasma triglyceride and cholesterol levels, which eventually trigger the development of lipotoxicity and lipid accumulation in the liver^10^. Fat accumulation in the liver is considered non-alcoholic fatty liver disease (NAFLD) and further progresses to steatosis. A “two hit” theory has been proposed to understand high-fat diet-induced NAFLD development^36,37^. Fat accumulation, insulin resistance, oxidative stress and ultimately inflammation are the responsible factors and important components of this “two hit” theory^38^. In the present study, rats fed a high-fat diet showed lipid accumulation and increased infiltration of inflammatory cells in the liver. Moreover, myeloperoxidase activity, which is a component of infiltrating cells, mainly neutrophils, was also increased in the liver of high-fat diet-fed rats. Probiotics have been considered beneficial to prevent inflammation in the intestine and liver^39,40^. Yogurt supplementation for four weeks decreases the lipid profiles of human subject^41^. Moreover, a probiotic mixture ameliorates the increased lipid profiles and decreases inflammatory markers in subjects with NAFLD^42^. In the present study, liver histology revealed a potential effect of yogurt supplementation on alleviating hepatic steatosis and inflammation in high-fat diet-fed rats.

Hepatic oxidative damage and inflammation may also trigger extracellular matrix deposition and fibrosis in the liver. Rats fed a high-fat diet have been reported to develop hepatic fibrosis, which was prevented by the supplementation of antioxidant-rich food^10^. Free radicles are postulated to play key roles in the activation of hepatic stellate cells (HSCs) and produce extracellular matrix, mainly collagen-type proteins. Local immune cells, mainly Kupffer cells, also stimulate the function of hepatic stellate cells to further increase the production of extracellular matrix proteins. Kupffer cells activate HSCs by producing the profibrotic cytokines TGF-β and platelet-derived growth factor (PDGF)^43^. Increased levels of endotoxins reach the liver via the portal vein and accelerate hepatic inflammation and fibrosis in animal models and patients with chronic liver diseases, emphasizing the relevance of gut-liver interactions^44^. Patients with liver cirrhosis show an enrichment of potentially more invasive bacterial strains in the gut microbiota^45^.

The intestinal epithelium possesses a mucosal barrier that limits inflammation attributed to the presence of microbes or other allergens in the intestine. Some lactic acid bacteria (LAB) reside in the gastrointestinal tract (GIT). These bacteria appear to exert beneficial effects on limiting the growth of pathogenic bacteria, maintaining intestinal permeability, regulating cytokine production, and regulating the immune response to some extent^46^. Probiotic supplementation reduces plasma cholesterol and triglyceride levels in high-fat diet-fed rats and humans^47^. The results from the present investigation are also consistent with the findings from previous studies and showed a decrease in plasma cholesterol and triglyceride levels. Bile acid deconjugation by probiotics and cholesterol binding have also been proposed as probable mechanisms underlying the cholesterol-lowering effect of probiotics^48^. Some *Lactobaccillus* species and other bacteria are capable of hydrolyzing the bile acid conjugates to make them water insoluble and eventually eliminate them through the feces^49^. This process increases the bile acid synthesis from cholesterol through homeostasis and inhibits the saponification of fatty acids to decrease fatty acid absorption from the gut^48^. Both processes decrease the plasma fatty acid levels available for cholesterol synthesis. Moreover, probiotics may convert cholesterol into coprostanol, which is directly excreted in feces^50^.

In the present study, the intestinal brush border was altered and a disorganized structure of the intestine was observed in rats fed a high-fat diet. The crypt region contained fewer goblet cells and fibrosis was observed in high-fat diet-fed rats. Yogurt supplementation normalized the intestinal microvilli and restored the crypt structure in high-fat diet-fed rats. This finding is also consistent with a previous report showing that probiotics effectively prevent intestinal inflammation and preserve the intestinal microstructure in high-fat diet-fed animals^38^.

In conclusion, this investigation provides evidence that yogurt supplementation is an alternative approach to combat obesity-related complications. Additionally, the consumption of a high-fat diet might alter the normal gut environment, and these changes can be modified by probiotic-rich yogurt supplement. Further research is warranted to confirm the beneficial effect on human subjects in a clinical trial.

## Materials and Methods

### Chemicals

Thiobarbituric acid (TBA) was purchased from Sigma-Aldrich (Germany). Reduced glutathione (GSH) was obtained from J.I. Baker (USA). Aspartate aminotransferase (AST), alanine aminotransferase (ALT), alkaline phosphatase (ALP), triglyceride, cholesterol, HDL and LDL assay kits were procured from DCI Diagnostics (Budapest, Hungary). All other chemicals, solvents and reagents used in the present study were of laboratory analytical grade.

### Yogurt (Sour Curd)

Yogurt (sour curd) from Aarong Dairy, Dhaka, Bangladesh was purchased from a local market in Dhaka, Bangladesh. The composition and preparation techniques of yogurt are provided in a supplementary file.

### Experimental animals and diet

The Ethics Committee of North South University approved all experimental protocols for animal care, handling and experimentation (AEC 008-2017). We would also like to confirm that all experiments were performed in accordance with relevant guidelines and regulations. Twenty four Wistar male rats (10–12 weeks old) weighing 175–195 g were obtained from the Animal Reproduction Unit of the Animal House of Department of Pharmaceutical Sciences, North South University. They were also housed in individual cases in an air-conditioned room (22 ± 2 °C) with 55% humidity on a 12 hr light/dark cycle and had free access to laboratory feed and pure water.

Normal laboratory food and a high-fat diet (HF) were used in this study. The normal food was composed of wheat, wheat bran, rice polishing and fish meal, with approximately 25% proteins, 60% carbohydrates and 15% fat in terms of the caloric content^51^. On the other hand the high-fat (HF) diet was composed of normal food, beef tallow, sugar and condensed milk and contained approximately 14% proteins, 37% carbohydrates and 49% fat in terms of the caloric content.

### Experimental design, animal sacrifice and sample collection

The experimental rats were divided into four groups, each comprising six rats:

- Control (Group 1), received normal water and normal food (powder) for eight weeks.
- HF (Group 3), received the HF diet for eight weeks.
- Control + Yogurt (Group 2), received normal water and normal food (powder) along with yogurt (5% w/w) for eight weeks.
- HF + Yogurt (Group 4), received the HF diet and yogurt (5%, w/w) for eight weeks.

The HF diet was prepared in our laboratory by mixing the powdered food, fat and carbohydrates (the formula is provided in the supplementary file). An OGTT was performed in rats from all four groups before and after completing the experimental feeding period to assess the glycemic activity before and after the consumption of the HF diet.

Body weight, food intake and water intake were recorded daily for 56 days. After 56 days, all animals were weighed and sacrificed by injecting pentobarbital anesthesia (90 mg/kg) in the peritoneal region. Blood samples were collected from the abdominal aorta into tubes containing citrate buffer at 4 °C. Within 30 min of the blood collection, blood samples were centrifuged at 8000 rpm for 15 min at 4 °C to separate the plasma. Separated plasma was transferred to 1.5 mL Eppendorf tubes and stored at −20 °C until further analysis. All other internal organs, including the heart, adipose tissues, spleen, kidney, intestine, and liver, were immediately collected after sacrificing the animals; the organs were also weighed and stored in neutral buffered formalin (pH-7.4) for histological analyses and stored at −20 °C for further biochemical studies.

### Oral glucose tolerance test

The oral glucose tolerance test was performed in all groups at the beginning of the experiments, followed by a 12 hrs fasting. The fasting blood glucose level was monitored in all rats using a commercial glucometer. A 2 g/kg dose of the glucose solution was administered and blood glucose level was monitored every 30 min for 120 min. At the end of the 56 day feeding period, rats were fasted for 12 hrs and an oral glucose tolerance test was performed as described previously^10^.

### Plasma biochemistry

Within 30 min of the collection of blood samples, blood was centrifuged at 8000 rpm for 15 min at 4 °C. Plasma was separated, transferred to 1.5 mL Eppendorf tubes and stored at −20 °C until analysis. Plasma concentrations of total cholesterol, HDL, LDL, and triglycerides and activities of aspartate transaminase (AST), alanine transaminase (ALT) and alkaline phosphatase (ALP) were determined using Diatec diagnostic kits (Hungary) according to the standards and protocols provided by the manufacturer.

### Preparation of tissue samples and analysis of oxidative stress markers

Liver tissue (one gram) was homogenized in 10 mL of phosphate buffer (pH 7.4) and centrifuged at 8000 rpm for 15 min at 4 °C. The supernatant was collected; protein and enzymatic analyses were performed using this supernatant. Lipid peroxidation in the liver was estimated by calorimetrically measuring thiobarbituric acid reactive substrates (TBARS) using a previously described method^52^. The absorbance of the clear supernatant was measured at 535 nm and normalized to a blank reference. Nitric oxide (NO) levels were measured as nitrate concentrations using the method reported by Tracey *et al*.^53^. The absorbance was measured at 540 nm and normalized to the corresponding blank solutions. The NO level was calculated from a standard curve and reported as nmol/g of tissue. APOP levels were determined using the methods described by Witko-Sarsat *et al*.^54^ and Tiwari *et al*.^55^, with some modifications. The chloramine-T absorbance recorded at 340 nm was linear within the range of 0 to 100 nmol/ml, and AOPP concentrations were reported as nmol.ml^−1^ chloramine-T equivalents.

### Assay of the activities of the antioxidant enzymes catalase activity assay (CAT) and superoxide dismutase (SOD) activity and reduced glutathione assay (GSH)

CAT activities were determined using a previously described method^56^. Changes in absorbance of the reaction solution at 240 nm were determined after 1 min. One unit of CAT activity was defined as a change in the absorbance of 0.01 unit/min. SOD activity was assayed in tissue homogenates and plasma using a previously described method^57^. Changes in the absorbance at 480 nm were recorded at 15 s intervals for 1 min. A control reaction consisting of all the ingredients except the enzyme was analyzed simultaneously. One unit of enzyme activity was defined as the 50% inhibition of the antioxidant activity of epinephrine present in the assay system. Reduced glutathione levels were estimated using the method reported by Jollow *et al*.^58^. After the yellow color developed, the absorbance of the mixture was immediately recorded at 412 nm using a Smart SpecTM plus spectrophotometer and reported as ng/mg protein.

### Estimation of myeloperoxidase (MPO) activity

MPO activity was determined using an *o*-dianisidine-H_2_O_2_ method modified for 96-well plates described by Rahman *et al*.^51^.

### Histopathological analysis

After sacrificing each rat, liver tissues were harvested and fixed with neutral buffered formalin for microscopy. The liver tissues were embedded in paraffin, sectioned at a 5 µm thickness and stained with hematoxylin/eosin to visualize the architecture of hepatic tissue and inflammatory cell infiltration. Sirius red staining was also performed to detect fibrosis detection, and Prussian blue staining was performed to analyze iron deposition in tissues. Moreover, periodic acid Schiff (PAS) staining was performed to identify the goblet cells in the intestine. Then, stained sections were examined and photographed under a light microscope (Zeiss Axioscope) at 40X magnification. At least 15 liver sections from three different animals in each group were analyzed and graded for hepatic lesions and fat deposition. ImageJ software, which is freely available from the National Institutes of Health (NIH), was used to measure the percentage of fibrosis in the liver and intestinal sections from each group.

A semiquantitative evaluation of NAFLD activity scores was proposed by the Pathology Committee of the NASH Clinical Research Network^59^. The guidelines and recommendations of the Committee based on the histological features are described as follows: steatosis (<5% = 0; 5–33% = 1; 33–66% = 2; >66% = 3); lobular inflammation (none = 0; 2 foci = 1; 2–4 foci = 2; >4 foci = 3); and hepatocellular ballooning (none = 0; few = 1; prominent = 2). All features were scored in a blinded manner in at least three rats from each group and five fields of view in each sample.

### Statistical analysis

All values are presented as means ± standard errors of the means (SEM). The results were evaluated using one-way ANOVA followed by the Newman-Keuls post hoc test with Graph Pad Prism Software (USA). Statistical significance was established at P < 0.05 in all experiments. N = 6. Asterisk (*) marked data are significantly different at p < 0.05 and (**) denotes p > 0.01. N = 6. Also, a vs b and c vs a or b are significantly different.

## Supplementary information

Supplementary information
