# Supplementary material for: High-fat diet-induced metabolic syndrome and oxidative stress in obese rats are ameliorated by yogurt supplementation
Source: Sci Rep. 2019 Dec 27;9:20026. doi: 10.1038/s41598-019-56538-0 (PMC6934669; doi:10.1038/s41598-019-56538-0)
Supplement: Supplementary file 1 — Supplementary information [file 41598_2019_56538_MOESM1_ESM.docx]

**Supplemental File**

**High-fat diet-induced metabolic syndrome and oxidative stress in obese rats are ameliorated by yogurt supplementation**

Shoumen Lasker^1^, Md Mizanur Rahman^1^, Faisal Parvez^1^, Mushfera Jamila^1^, Pintu Miah^1^, Kamrun Nahar^1^; Fariha Kabir^1^; Surovi Binte Sharmin^1^, Nusrat Subhan^1^, Gias U. Ahsan*^2^,

and Md Ashraful Alam*^1^

^1^Department of Pharmaceutical Sciences, North South University, Dhaka- 1219, Bangladesh.

^2^Department of Public Health, School of Health and Life Sciences, North South University, Bangladesh.

**Materials and Methods:**

**Yogurt preparation and composition:**

This yogurt is made up of cow’s milk, skimmed milk powder and bacterial cultures. Mainly two bacterial cultures were used to prepare the yogurt, such as *Streptococcus lactis* and *Lactobacillus bulgaricus. Y*ogurt also contains 3.2 g fat, 5.03 g carbohydrate, 5.67 g protein and 169 mg calcium in 100 g which can provide 71.6 Kcal energy. In general, collected milk was pasteurized first at 90º C for 16 seconds and then transferred to yogurt milk processing tank where bacterial cultures were added at 41-43º C temperature. This processed milk is then transferred to small containers where the yogurt was appeared at incubation temperature of 43ºC. The prepared yogurt containers were kept under 4º C temperature in storage condition.

**Table 1:** Composition of normal and high-fat diet used in this study (for 100 g).

| **Control diet** | **%** | **HF diet** | **%** |
| --- | --- | --- | --- |
| Wheat | 40% | Powdered normal rat feed | 15.5% |
| Wheat Bran | 20% | Sugar | 17.5% |
| Rice Polishing | 0.5% | Beef tallow (fat) | 20.0% |
| Fish meal | 1.0% | Condensed milk | 39.5% |
| Oil cake | 1.0% | Embavit (vitamin mixture) | 0.1% |
| Gram | 0.39% | Salt | 0.5 % |
| Pulses | 0.39% | Water | 100 ml |
| Milk | 0.38% |  |  |
| Soybean Oil | 0.15% |  |  |
| Molasses | 0.095% |  |  |
| Salt | 0.095% |  |  |
| Embavit (vitamin mixture) | 0.1% |  |  |

Chow diet contained as percentage of calories 14% proteins, 57% carbohydrates, 13.5% fat. High carbohydrate high fat diet contained as percentage of calories 14% proteins, 37 % carbohydrates, 48 % fat.

**Results:**

**Prussian blue Staining:**

**
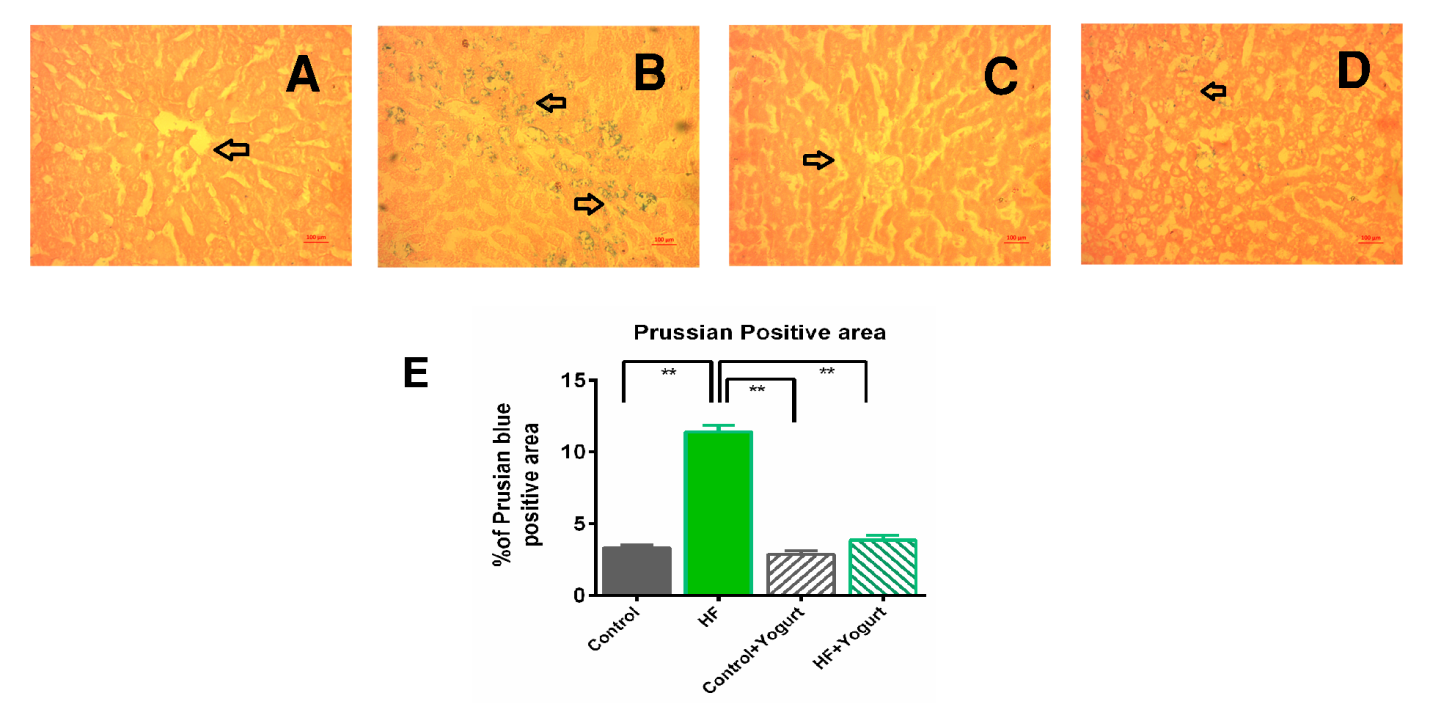
**

**Figure 1:** Effect of yogurt supplementation on iron droplet deposition in liver of high fat (HF) diet fed rats. Control rats were provided with control diet and HF rats were provided with HF diet. Yogurt was also supplied to control+yogurt and HF+yogurt groups. A-Control, showed no Prussian blue dot in the liver section. B-HF showed Prussian blue positive area in the liver section as a sigh on free iron deposition. C- Control+yogurt, showed no Prussian blue dot in the liver section as in control rats. D- HF+Yogurt, yogurt supplementation prevented the iron deposition in liver section of HF diet fed rats. Magnification is X40. E- percentage of Prussian blue positive area in various groups of rats. Data are presented as mean±SEM. Statistical analysis was performed by One Way ANOVA with Newman-Keuls post hoc test. Statistical significance is considered as p<0.05. Asterisk (*) marked data are significantly different at p<0.05 and (**) denotes p>0.01.
